# Molecular characterization of *Anaplasma* and *Ehrlichia* in ixodid ticks and reservoir hosts from Palestine: a pilot survey

**DOI:** 10.1002/vms3.150

**Published:** 2019-02-14

**Authors:** Taher Zaid, Suheir Ereqat, Abdelmajeed Nasereddin, Amer Al‐Jawabreh, Ahmad Abdelkader, Ziad Abdeen

**Affiliations:** ^1^ Biochemistry and Molecular Biology Department Faculty of Medicine Al‐Quds University Abu Dis Palestine; ^2^ UCD School of Veterinary Medicine University College Dublin Belfield Ireland; ^3^ Al‐Quds Public Health Society East Jerusalem Palestine; ^4^ Arab American University Jenin Palestine

**Keywords:** *Anaplasma*, dogs, *Ehrlichia*, Ixodid ticks, Palestine, sheep

## Abstract

Tick‐borne anaplasmosis and ehrlichiosis are clinically important emerging zoonoses usually overlooked by veterinarians and physicians alike. This study aimed at detecting and genetically characterizing *Ehrlichia* and *Anaplasma* species in ixodid ticks and their animal hosts from the West Bank, Palestine. A total of 723 ixodid ticks belonging to three genera (*Rhipicephalus, Hyalomma, Haemaphysalis*) were collected from dogs, sheep, goats and camels. In addition, 189 blood samples were collected from dogs, sheep, camels, horses and a goat from the West Bank, Palestine. All tick and blood samples were investigated for the presence of *Anaplasma* and *Ehrlichia* targeting a 345 bp fragment of the 16S rRNA gene followed by sequence analysis. The infection rate of *Anaplasma* spp. in ticks was 6.5% (47/723). *Anaplasma platys* was identified in 28% (13/47) of them. Whereas, based on a partial sequence (851 bp) of *msp4* gene, 38% (18/47) were identified as *A. ovis*. The species of the remaining 16 positive samples (16/47, 34%) could not be identified. Simultaneously, the infection rate of *Ehrlichia* spp. in the ticks was 0.6% (4/723). Three of which were *E. canis* and one was *Ehrlichia* spp. The infection rate of *A. platys* in dogs’ blood samples was 10% (13/135), while it was 1.5% (2/135) for *E. canis*. The infection rate of *Anaplasma* in sheep blood samples was 40% (19/47), out of which 26% (5/19) were caused by *A. ovis* as revealed by *msp4*‐PCR. Implementation of purely‐spatial analysis by saTScan for all cases of *Anaplasma* revealed two statistically significant clusters in two districts; Tubas town and Majdal‐Bani‐Fadil village on the western hills of the Jordan Valley. Most cases of *Anaplasma* (83%) were from rural areas where life cycle components (vector, host and reservoir) abundantly interact. This study is the first in Palestine to reveal the presence of *Anaplasma* and *Ehrlichia* in ticks, dogs and sheep providing crucial platform for future epidemiological surveys and control strategies in the country and region.

**Impacts**

- Among neglected tick‐borne bacterial zoonoses in Palestine, ehrlichiosis and anaplasmosis are considered emerging diseases worldwide with increasing number of human cases and substantial economic burden.
- In Palestine and for the first time, anaplasmosis and ehrlichiosis have been detected and genetically characterized in ixodid ticks and their animal hosts.
- The study broached the possibility of cross‐border spill out of infection to neighboring countries across Jordanian and Israeli borders which should trigger regional cooperation to control the diseases.

## Introduction

In Palestine and elsewhere in the world, ehrlichiosis and anaplasmosis are considered neglected tick‐borne bacterial zoonoses caused by *Ehrlichia* and *Anaplasma*. The two genera belonging to the family Anaplasmataceae encompass groups of obligatory intracellular Gram‐negative bacteria invading blood cells of mammals including leukocytes, erythrocytes and thrombocytes (Yang *et al*. 2015). The reservoir hosts include numerous wild and domesticated animals (Dumler *et al*. 2001; Ismail *et al*. 2010). Hard ticks from the Ixodidae family usually transmit these pathogens to mammals; in addition, they can be transmitted directly to both human and animals by blood transfusion (Fine *et al*. 2016; Marenzoni *et al*. 2017). The genus *Ehrlichia* contains six recognized species: *Ehrlichia canis, E. chaffeensis, E. ewingii, E. muris, E. ruminantium and E. minasensis* (Cabezas‐Cruz *et al*. 2016). The currently recognized six species in the genus *Anaplasma* are *Anaplasma phagocytophilum* which cause human granulocytic anaplasmosis (HGA), *A. platys, A. marginale, A. bovis, A. ovis*,* A. capra* and *A. odocoilei* (Dumler *et al*. 2001; Ndip *et al*. 2010; Tate *et al*. 2013; Li *et al*. 2015; Silaghi *et al*. 2017). Ixodid ticks maintain different *Anaplasma* species in nature. Various species of *Ixodes*,* Rhipicephalus, Amblyomma* and *Dermacentor* serve as vectors for *Anaplasma* spp. worldwide including neighboring countries as Egypt and Israel (Loftis *et al*. 2006a,b; Harrus *et al*. 2011). *R. sanguineus s.l*. is a recognized vector of different pathogenic agents including bacteria, protozoa, nematodes and viruses that affect dogs and infrequently humans (Dantas‐Torres & Otranto 2015).

The clinical manifestations of ehrlichiosis and anaplasmosis are similar in both human and animals. The consequences of infection vary from asymptomatic infections or mild symptoms to a severe, potentially, fatal illness. In human, these diseases are characterized by fever, headache, chills and muscle aches within two weeks of the tick bite which is often accompanied by thrombocytopenia, leukopenia and elevated levels of hepatic enzymes in the blood (Ismail *et al*. 2010). Cattle can be infected by several *Anaplasma* species, like *A. marginale*,* A. phagocytophilum*,* A. centrale* and *A. bovis* (Silaghi *et al*. 2017). *A. marginale* is known to be highly pathogenic in cattle, whereas *A. centrale* is less virulent and is being used for immunization against anaplasmosis (Aubry & Geale 2011). *A. ovis* is moderately pathogenic in sheep, goats, and wild ruminants and causes acute disease in animals exposed to stress, hot weather, deworming and animal movement (Kuttler 1984; Friedhoff 1997). In dogs, different pathogenic *Anaplasma* and *Ehrlichia* species have been reported with ehrlichiosis showing generally more severe symptoms than anaplasmosis (Ismail *et al*. 2010; Sainz *et al*. 2015). Canine monocytic ehrlichiosis (CME) is a systemic infection in dogs caused by *E. canis*. Its clinical symptoms may vary but include fever, weight loss, lethargy, lymphadenopathy, splenomegaly, hepatomegaly, thrombocytopenia, bleeding disorders, bone marrow failure and may lead to death in dogs and other canids (Ismail *et al*. 2010; Sainz *et al*. 2015).

*A. platys* infection in dogs is reported to be either with few or no clinical signs or more virulent, while dogs infected with *A. phagocytophilum* may remain healthy or manifest clinical signs including fever, lameness, lethargy and anorexia (Ismail *et al*. 2010; Sainz *et al*. 2015). In camels, recent studies reported the presence of *Anaplasma* and *Ehrlichia* spp. (Sudan *et al*. 2014; Bastos *et al*. 2015). Although decision for treatment can be based on clinical signs and symptoms, yet microscopic examination of Giemsa‐stained thin peripheral blood smears was used to demonstrate cytoplasmic morula for diagnosis of ehrlichiosis and anaplasmosis. However, this method is only useful for detecting clinically suspected animals during the acute phase of the disease thus reducing sensitivity (Paddock & Childs 2003; Ismail *et al*. 2010; Bakken & Dumler 2015). Currently, serological methods with Immunofluorescence antibody (IFA) assay as the gold standard and enzyme‐linked immunosorbent assay (ELISA) method are used to diagnose both infections (CDC, 2008). However, cross‐reactivity between genera and species has been reported (Al‐Adhami *et al*. 2011). Therefore, molecular‐based methods such as polymerase chain reaction (PCR) and real‐time PCR, targeting different genes have been developed to detect and identify *Anaplasma* spp. and *Ehrlichia* spp. with fairly higher sensitivity and specificity (Parola *et al*. 2000; Dong *et al*. 2013). Several *Anaplasma* strains have been detected using the small‐subunit rRNA (16S rRNA) which has proven to be a sensitive molecular tool to confirm the presence of these pathogens’ DNA in the investigated ticks and/or animal hosts. However, 16S rRNA gene is highly conserved with few polymorphic positions, therefore, closely related species and strains cannot be distinguished (Mongruel *et al*. 2017). On the other hand, it has been reported that the major surface protein 4 (msp 4)‐that is encoded by *msp4* gene‐is associated with faster evolution than other nuclear genes and involved in interactions with host cells (Yang *et al*. 2015). Thus, the genetic diversity of *msp4* sequences is useful to reveal intraspecies variation and phylogenetic studies of several *Anaplasm*a strains obtained from different hosts (Paulauskas *et al*. 2012).

Despite being emerging diseases worldwide with increasing number of human cases and the substantial economic burden with livestock infection, anaplasmosis and ehrlichiosis have not been investigated and no previous data is available on any of these diseases in Palestine. Therefore, this study was conducted to (i) detect and genetically characterize *Anaplasma* and *Ehrlichia* spp. in hard ticks and blood samples collected from domestic dogs, sheep and camels (ii) determine geographical distribution of *Anaplasma* and *Ehrlichia* spp. in the West Bank, Palestine.

## Methods

### Study design

**Figure 1:**
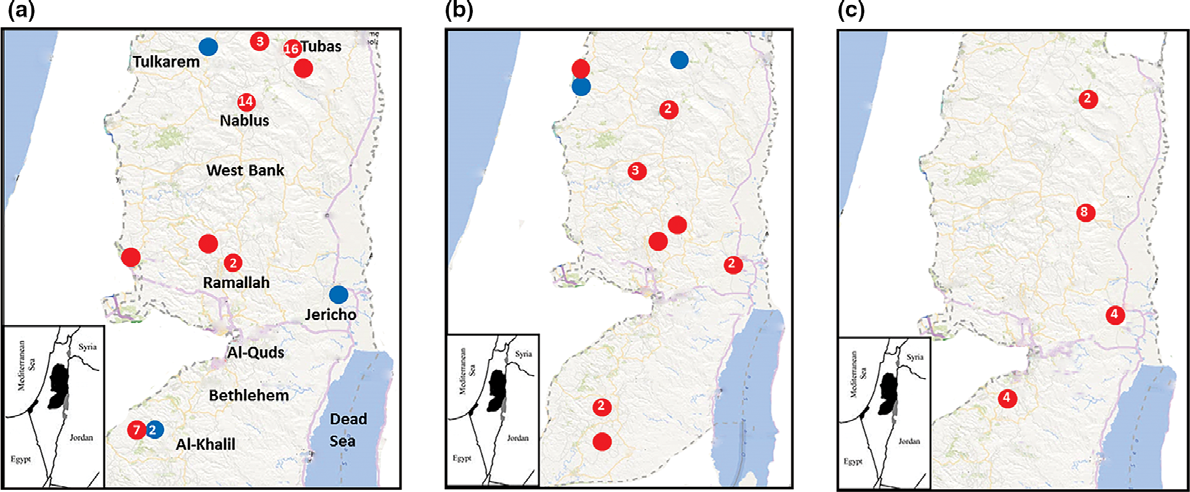
Spot maps of cases of *Anaplasma* (red circles) and *Ehrlichia* (blue circles) isolated from ticks (a), dogs (b) and sheep (c). The number within the circles indicates cases spotted in the area while those without numbers indicate one case.

A total of 723, partially engorged, hard ticks were collected during January to April, 2015 from 253 animals including dogs, camels, sheep and goats in nine districts (Jenin, Tubas, Tulkarm, Nablus, Jericho, Ramallah, Salfit, Bethlehem and Al‐Khalil) located in three zones in the central, northern and southern regions of the West Bank‐Palestine representing the overall tick population in the country (Fig. 1a, Table 1). At the time of tick sampling, blood samples were collected from different outdoor domestic dogs (*n* = 135 and camels (*n* = 4). In 2016, additional blood samples were taken from sheep (*n* = 47), horses (*n* = 2) and goats (*n* = 1) from Jericho and Bethlehem. Study animals were selected randomly; all of them were apparently healthy and did not show any clinical signs at the time of sampling.

**Table 1:** Overall infection rate of *Anaplasma* and *Ehrlichia* infections in ticks using 16SrRNA PCR

*Ixodid* tick species	# of tested ticks	Positives (%)	Life stage (*n*)	Animal hosts	District	Pathogen detected (*n*)
*Rhipicephalus sanguineus*	508	26 (5.1)	Female (16) Male (5) Nymph (5)	Dogs, Sheep, Goats	Jenin, Ramallah, Al‐Khalil, Tubas, Nablus, Jericho, Tulkarm	*Anaplasma platys* (13)*, Anaplasma* spp (10), *Ehrlichia canis* (3)
*Rhipicephalus turanicus*	108	21 (19.4)	Female (12) Male (9)	Sheep, Dogs	Nablus, Tubas, Jenin, Ramallah, Jericho.	*Anaplasma* spp (21)
*Rhipicephalus bursa*	11	0		Sheep, Goat, Dogs	Tubas, Jenin, Ramallah	
*Rhipicephalus* spp.	32	3 (9.4)	Female (3)	Sheep, Dogs	Nablus, Jenin, Ramallah, Al‐Khalil, Tubas, Jericho	*Anaplasma* spp (3)
*Hyalomma dromedarii*	32	0				
*Hyalomma impeltatum*	5	0		Camels	Jericho	
*Hyalomma* spp.	6	0				
*Haemaphysalis parva*	16	1 (6.3)	Female (3)	Dogs	Ramallah, Jenin, Tulkarm	*Ehrlichia* spp (1)
*Haemaphysalis adleri*	5	0		Dogs	Jenin, Ramallah	
Total	723	51 (7.1)				

### Ticks and blood samples

Two to five hard ticks were taken per animal host. All ticks were microscopically identified to the genus and species level using standard taxonomic keys (Guglielmone *et al*. 2009). Tick samples were separated into different micro‐centrifuge tubes containing 70% alcohol and kept at −20°C until DNA extraction. Blood samples (*n* = 189) were collected from different domestic animals including, dogs, sheep, camels, horses and a goat. All blood samples were collected in EDTA‐anticoagulant tubes and stored at −20°C until further use.

### DNA extraction

Prior to DNA extraction, individual ticks were washed with phosphate‐buffered saline (PBS), air‐dried for 10 min on tissue paper and separately sliced into small pieces by a sterile scalpel, then manually homogenized with a sterile pestle and mortar. The sliced sample was re‐suspended in 200 *μ*L of lysis buffer and 20 *μ*L of proteinase K followed by DNA extraction using QIAamp animal blood and tissue Kit procedure (Qiagen GmbH, Hilden, Germany). DNA was extracted from whole blood samples (300 *μ*L) using the same kit mentioned above. DNA concentration was measured by Nanodrop (Thermo Scientific NanoDrop 1000) and kept frozen at −20 until further use.

### PCR amplification and DNA sequence analysis

All DNA samples were screened by PCR using a primer pair, EHR16SR (5′‐ TAGCACTCATCGTTTACAGC‐3′) and EHR16SD (5′‐GGTACCYACAGAAGAAGTCC‐3′, targeting a 345 bp fragment of the 16S rRNA gene. These primers are specific for the family Anaplasmataceae, including the genera *Anaplasma, Ehrlichia, Neorickettsia* and *Wolbachia* (Parola *et al*. 2000). PCR was performed as described previously (Parola *et al*. 2000) with the following modifications; the PCR reactions were performed in a total volume of 25 *μ*l using PCR ready mix (Thermo Fisher Scientific) containing 1 *μ*mol/L of each set of primers and 5 *μ*l of the extracted DNA.

### Identification of *Anaplasma* spp. targeting *msp4* gene

A fragment of 851 bp of the major surface protein gene (*msp4*) was amplified and sequenced to differentiate between *A. marginale, A. centrale* and *A. ovis* using the previously published primers (MSP45 (5′‐GGGAGCTCCTATGAATTACAGAGAATTGTTTAC‐3′) and MSP43 (5′‐CCGGATCCTTAGCTGAACAGGAATCTTGC‐3′)(de la Fuente *et al*. 2003). The *msp4*‐PCR reactions were performed in 25 *μ*L PCR‐ready supreme mix (Syntezza Bioscience‐Jerusalem), containing 1 *μ*mol/L of each set of primers and 5 *μ*L of the extracted DNA. The thermal cycling procedure was as described previously (de la Fuente *et al*. 2003).

In all amplification reactions, negative controls (without DNA) were included. PCR amplifications were carried out in BiometraT Professional basic 96 gradient thermocycler. PCR products were visualized under UV illumination after electrophoresis on 2% agarose gels stained with ethidium bromide using different DNA ladders (100 bp and 1 kb) as molecular markers (Thermo Scientific GeneRuler). Gels were captured using Minilumi machine (DNR Bio Imaging Systems ltd).

### DNA sequence analysis

Forward and reverse sequences of all amplified PCR products were obtained. The sequences were analyzed and evaluated with The Sequence Manipulation Suite program (Stothard 2000) and multiple sequence alignment with hierarchical clustering (http://multalin.toulouse.inra.fr/multalin/) (Corpet 1988). Species identity for DNA sequences was assessed based on the closest BLASTn match (identity ≥ 99%) using the MegaBLAST with homologous sequences deposited in NCBI database (National Center for Biotechnology Information, U.S. National Library of Medicine, 8600 Rockville Pike, Bethesda MD). CLUSTALW program (http://www.genome.jp/tools/clustalw/) was used for the multiple sequence alignment. Phylogenetic trees construction was carried out using the statistical method Maximum Likelihood (ML) with bootstrap of 1000 replications using MEGA X program (Kumar *et al*. 2018). Partial DNA sequences of 16S rRNA (345 bp) and *msp4* genes (851 bp) were used to build the trees based on complete deletion option with gaps and missing data were eliminated. All based on Jukes‐Cantor model for nucleotide sequences. Initial trees for the heuristic search were automatically obtained by applying the Nearest‐Neighbor‐Interchange (NNI) algorithms to a matrix of pairwise distances estimated using the Maximum Composite Likelihood (MCL) approach. The DNA sequence of *Neorickettsia sennetsu* (NR_044746.1) was used as an out‐group to produce a rooted tree.

### Statistical analysis

Frequency tables, distributions and rates (positive/total tested) were calculated using EpiInfo™ statistical package (CDC free‐software). SaTScan™ v8.0 Freeware was used to detect statistical evidence for purely‐spatial clustering of cases caused by *Anaplasma* spp. Analysis was done on two levels, the first included segregation of cases based on the host, while the second was based on pooling of all cases regardless of host. It's based on a scanning window that moves across space. For each geographical location, a hypothetical window is drawn with observed and expected number of cases. The cases inside the window are compared to those outside. The window with the greatest observed‐to‐expected ratio is spotted on the map. The window identified as the least likely due to chance is subsequently evaluated by a maximum likelihood ratio test with a test decision based on a Monte‐Carlo simulated *P*‐value (999 simulations). The maximum proportion of the population that a cluster could contain was set at 50% of the cases. Circles were restricted to 1 km radius with no central overlap with other clusters. Input files included number of cases per locality, year of infection and total number of tested samples. Data were analysed based on discrete Poisson model with level of statistical significance considered at *P*‐value ≤ 0.05 (Kulldorff 1997). Significant clusters of cases were spotted on maps using Epi Info 7 based on exact longitude‐latitude coordinates of each location.

## Results

### Ticks identification

The ticks comprised three genera *Rhipicephalus*,* Hyalomma* and *Haemaphysalis*. Among which, 508 were *R. sanguineus s.l* (240 females, 210 males, 57 nymphs and one with undefined life stage), 108 *R. turanicus* (60 females, 43 males and five nymphs), 11 *R. bursa* (six females and five males), 32 *Rhipicephalus* spp. (27 females, three males, one nymph and one with undefined life stage), 32 *Hy. dromedarii* (nine females and 23 males), five *Hy. impeltatum* (one female and four males), six *Hyalomma* spp. (five females and one male), 16 *H. parva* (11 females, three males and one nymph) and five *H. adleri* (all of them were females).

### Molecular detection of *Ehrlichia* and *Anaplasma* spp. in ticks

All samples (723 ticks) were screened for the presence of *Anaplasma* and *Ehrlichia* spp. DNA using 16S rRNA‐PCR. The sample was considered positive if a fragment of 345 bp was observed on 2% agarose gel. The infection rate for *Anaplasma* and *Ehrlichia* collectively in ticks was 7.1% (51/723). To identify the type bacteria; the amplified products were sequenced and subsequently matched with BLAST algorithm. Based on this, the infection rate of *Ehrlichia* spp. was 0.6% (4/723). The infection rate of *Anaplasma* spp was 6.5% (47 /723), of which 28% (13/47) were *A. platys* (as revealed by 16S rRNA PCR) and 38% (18/47) were *A. ovis* as revealed by *msp‐4* PCR. The species of the remaining 16 positive samples (16/47, 34%) could not be identified. The 13 *A. platys* were detected in *R. turanicus* (*n *= 8) and *R. sanguineus* (*n* = 5). Three DNA sequences obtained from *R. sanguineus s.l*. were identified as *E. canis,* whereas one sequence from *H. parva* belonged to *Ehrlichia spp*. The tick species and their animal hosts are shown in (Table 1).

### *Ehrlichia* and *Anaplasma* in blood samples

Of the 189 animal blood samples screened by 16S rRNA‐PCR, 34 samples (18%) were positive for *Anaplasma* or/and *Ehrlichia* (Table 2). None of the blood samples from camels (*n* = 4), horses (*n* = 2) and goats (*n* = 1) were positive (Table 2). Among the canine blood samples (*n* = 135), 11.1% (15/135) were positive (Table 2). The infection rate of *A. platys* in dogs’ blood was 10% (13/135) and 1.5% (2/135) for *E. canis*. The infection rate of *Anaplasma* in sheep blood samples was 40% (19/47) of which 26% (5/19) were caused by *A. ovis* as revealed by *msp4*‐PCR. *Ehrlichia* was not detected in sheep.

**Table 2:** Overall infection rate of *Anaplasma* and *Ehrlichia* infections in animal blood samples

Animal species	no. of animals	Positives (%)	District	Pathogens detected (*n*)
Dogs	135	15 (11.1)	Jenin, Al‐Khalil, Ramallah, Jericho, Salfit, Tulkarm, Nablus	*Ehrlichia canis* (2)*, Anaplasma platys* (13)
Sheep	47	19 (40.4)	Jericho, Bethlehem	*Anaplasma* spp. (19)
Camels	4	0	Jericho	
Goats	1	0	Bethlehem	
Horses	2	0	Jericho	
Total	189	34 (18)		

### Phylogenetic analysis

**Figure 2:**
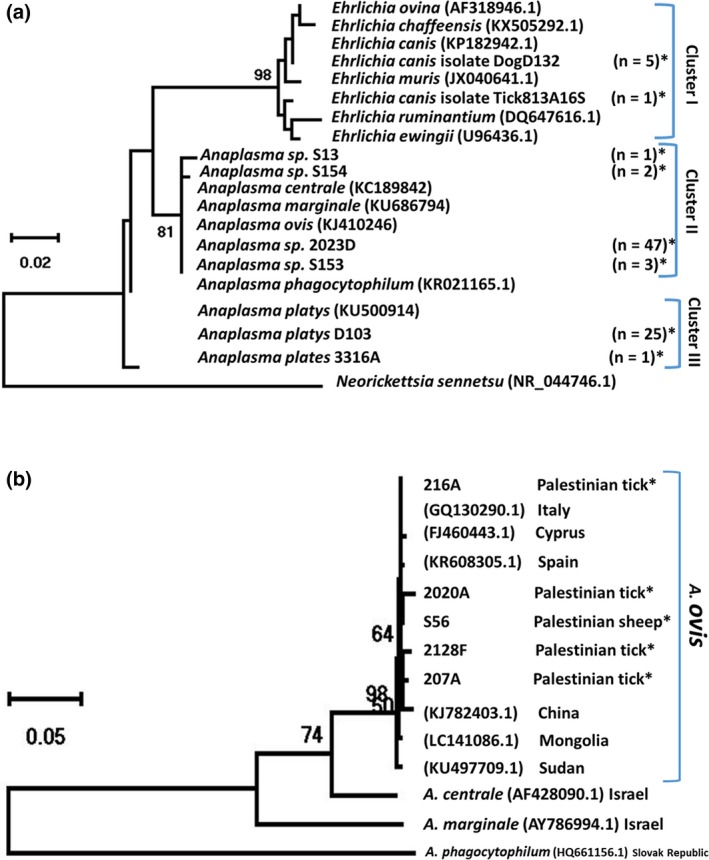
Phylogenetic analysis of *Anaplasma* and *Ehrlichia* based on partial sequences of (a) *16S rRNA* and (b) *msp4* genes. Phylogenetic analysis were constructed using the maximum likelihood method used on MEGA X program, with the complete deletion option, based on Jukes‐Cantor model for nucleotide sequences. Initial trees for the heuristic search were automatically obtained by applying the Nearest‐Neighbor‐Interchange (NNI) algorithms to a matrix of pairwise distances estimated using the Maximum Composite Likelihood (MCL) approach. Statistical support for internal branches of the trees was evaluated using bootstrap of 1000 replications. (a) Based on 16srRNA sequences (345 bp): cluster I represents *Ehrlichia* strains. Cluster II represents *Anaplasma* spp. and Cluster III represents *Anaplasma platys*. The DNA sequence of *Neorickettsia sennetsu* (NR_044746.1) was used as an out‐group to produce rooted tree. (b): 851 bp *msp4* DNA *Anaplasma* sequences detected in this study compared to *Anaplasma* reference sequences deposited in the NCBI GenBank. The Species, GenBank accession numbers and country of origin from which the sequences were derived are included for each sequence. Sequences derived from this study are marked by star (*). The number of identical sequences is in brackets. Selected reference *Anaplasma* spp. sequences from GenBank are also shown.

Phylogenetic analysis based on partial sequences of 16S rRNA gene revealed two main clusters: Cluster I represented the strains of *Ehrlichia* (*n* = 6). The DNA sequences from ticks (*n* = 3) and those from dogs (*n* = 2) were identical to each other and to the *E. canis* strain deposited in the Genbank (KP182942.1). One sequence from *H. parva* tick formed a separate branch and showed 99% sequence identity to the strain of *Ehrlichia* spp. (KJ410253.1) (Fig. 2a). Cluster II representing the strains of *Anaplasma* spp. (*n *= 53), shared at least 99% sequence identity to each other and to the sequences of *A. centrale, A. marginale and A. ovis* (KC189842.1, KU686794.1 and KJ410246.1, respectively) (Fig. 2a). Cluster III, represents the strains of *A. platys* (*n *= 26) were obtained from dogs (*n* = 13) and ticks (*n *= 13) and showed 99–100% sequence identity to each other and to the sequence of *A. platys* deposited in GenBank (KU500914.1). Phylogenetic analysis based on the amplification of partial sequences of *msp4* gene for different *Anaplama* spp. revealed all 18 *Anaplasma ovis* from Palestine grouping into one cluster (Fig. 2b). Representative sequences for 16S rRNA obtained in the course of this work were deposited into GenBank under the accession numbers of MK069487 and MK069495 for 16rRNA and under the accession numbers of MK087764 and MK087768 for *msp4* gene.

### Spot mapping and statistically significant clusters

**Figure 3:**
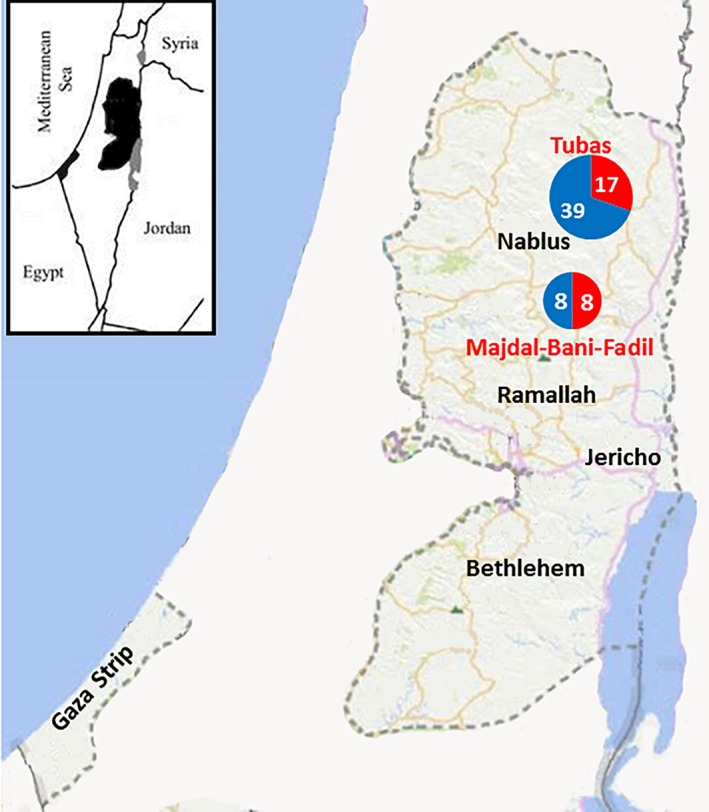
Geographical distribution of statistically significant foci of *Anaplasma* cases in Palestine (excluding Gaza strip) on implementing purely spatial analysis by SaTScan: red circles indicate positive cases while blue circles indicate negative cases. The numbers within the circles indicate the number of cases.

In the nine districts in the West Bank, 50 Palestinian localities targeted for the collection of samples. Cases of both infections in ticks, dogs and sheep in the study originated from 18 (36%) localities, which included villages (11/18 = 61%), cities (6/18 = 33%) and refugee camps (1/18 = 6%). Most of them (12/18 = 67%) were from rural areas. However, cities like Jericho, Tubas and Salfit are by standards considered towns rather than well‐established cities with rural activities on the margins. *Anaplasma* DNA was detected by 16S‐rRNA PCR in dogs in 10 (29%) out of 35 localities (Fig. 1b), while from ticks in eight (21%) localities out of 39 (Fig. 1a). At the same time, it was detected from sheep in all seven targeted localities (Fig. 1c). *Ehrlichia* DNA positive cases were from the districts of Al‐Khalil, Jericho, Tulkarem, and Jenin (Fig. 1a–c). Implementation of purely‐spatial analysis by saTScan for the pooled cases of *Anaplasma* from ticks, dogs and sheep revealed two statistically significant clusters (*P* < 0.05) (Fig. 3). However, implementing the same analysis on segregated bulks of cases did not reveal any significant clusters. Significant clusters were in Tubas town (*P* = 0.00005, Relative risk (RR) = 4.3) and Majdal‐Bani‐Fadil village near Ramallah (*P* = 0.0012, RR = 6.5) (Fig. 3). In Al‐Khalil district south of the West Bank, Hitta village close to the Green Line (1949 Armistice border between Palestinians and Israelis) had ten cases of *Anaplasma* infection which was close to forming a cluster (*P* = 0.08; RR = 3).

## Discussion

Tick‐borne bacteria are important pathogens which affect the health of both humans and animals globally. In this study and for the first time in Palestine, we have reported the presence of *Anaplasma* and *Ehrlichia* in ixodid ticks and blood samples from different domestic animals. These pathogens have been reported from different neighboring countries including; Egypt, Israel and Iran (Loftis *et al*. 2006a,b; Harrus *et al*. 2011; Jafarbekloo *et al*. 2014). Two pathogens were detected in canine blood samples: *A. platys* and *E. canis*. The prevalence of *A. platys* determined in this study (10%) was lower than Kenya (12.5%) (Matei *et al*. 2016) and Brazil (19.4%) (da Silva *et al*. 2012).

In congruence with a study reported from Japan, *E. canis* was detected in 1.5% of the tested canine blood samples (Kubo *et al*. 2015). In contrast, two studies conducted in Brazil (da Silva *et al*. 2012) and Panama (Santamaria *et al*. 2014) reported much higher prevalence; 16.4% and 64.2%, respectively. Despite that *E. canis* is well‐known as a dog pathogen; it has been reported in domestic ruminants (Zhang *et al*. 2015). However, in this study, *E. canis* was not detected in sheep, camels, goats and horses.

Furthermore*, A. platys* and *E. canis* were identified in Ixodid ticks obtained from the same infected dogs. Although the blood and tick samples were collected from dogs simultaneously, our results showed that the prevalence of *A. platys* in ticks (1.8%) was lower than in dogs (9.6%). The discrepancy in the prevalence could be attributed to unequal burden of tick populations per animal host, different structure of the tick community which is derived from dogs from nine districts in Palestine and possibility of more than one tick species acting as vector in a given area. Our findings showed that *A. platys*‐infected ticks were mainly from the species of *R. sanguineus s.l*. which has been reported as the most prevalent dog tick in Palestine (Dantas‐Torres 2008a,b; Ereqat *et al*. 2016a,b; Harrus *et al*. 2011). However, the presence of *A. platys* in other species of *Rhipicephalus*, such as unengorged *R. turanicus* and *R. bursa* were reported in Turkey and Israel (Aktas *et al*. 2009; Harrus *et al*. 2011). Additionally, the prevalence of *E. canis* in ticks (0.6%) was lower than in dogs (1.5%). Higher infection rates of *E. canis* in tick were reported from Iran (16.7%) (Khazeni *et al*. 2013) and Israel (10%) (Harrus *et al*. 2011). However, in that study, questing ticks have been collected from vegetation by flagging while engorged ticks were collected from dogs in our study. Leschnik *et al*. (2012) demonstrated that sampling strategy for collecting ticks and the time of collection affect the species composition of the sample, developmental stages and the prevalence of their microbial pathogens (Leschnik *et al*. 2012). Moreover, co‐occurrence of infected nymphs and susceptible larvae on the same host and spatial clustering of ticks on the same host surfaces appear to be essential for transmission from one tick to another which may influence the prevalence of microbial pathogens in the ticks (Leschnik *et al*. 2012).

On the basis of 16SrRNA phylogenetic analysis, similarity was observed among the sequences of *A. platys* identified in this study. Furthermore, no heterogeneity was observed among *E. canis* group using 16S rRNA gene. However, distinct Ehrlichia sequence was found to be 99% similar to the corresponding sequence of a not well identified *Ehrlichia* spp. reported from China (Dong *et al*. 2014; Kang *et al*. 2014). Further characterization with additional genes is needed to reveal the species.

In this study, *A. ovis*, the agent of ovine anaplasmosis, was identified for the first time in Palestine. The overall prevalence of *A. ovis* in sheep (26%) and their corresponding ticks (38%) was lower than reported by other studies conducted in northwest China (Yang *et al*. 2015), Iraq, Sudan, Portugal and Turkey (Renneker *et al*. 2013). However, the blood samples were not taken at the same time as the ticks.

Our findings provide molecular evidence for the presence of *A. ovis* in *R. turanicus* and *R. sanguineus s.l*. ticks which was in line with previous studies, showing that *Rhipicephalus* spp. is one of the most important vectors of diseases in sheep (Renneker *et al*. 2013; Hosseini‐Vasoukolaei *et al*. 2014; Jafarbekloo *et al*. 2014).

Amplification of 16SrRNA gene is commonly used for the detection of *Anaplasma*/*Ehrlichia* DNA and thus further testing is required to investigate co‐infections by the two pathogens and for species identification of the same genus such as *A. marginale* and *A. ovis*. In the present study, and since 16SrRNA PCR was unable to definitively identify most of the *Anaplasma* species*; A. ovis* infections have been identified using *msp4* primers (de la Fuente *et al*. 2003).

The presence of *A. ovis* is confirmed in Palestine as around the world, although this bacterium is supposed to cause only mild clinical symptoms, its adverse effect if the animals were under stress by different factors such as poor health conditions, hot weather, co‐infection, heavy tick infestation, vaccination or deworming is aggravated in infected ruminants (Hornok *et al*. 2007). Since the small ruminants are major source of meat, milk and wool in Palestine, ovine anaplasmosis, caused by *A. ovis*, may lead to economic burden if stress occurs at any time, so it is important to better understand this disease and further investigations are necessary.

Spot mapping of cases confirmed that most of the cases of anaplasmosis and ehrlichiosis (83%) were in rural areas where the vast majority of livestock and accompanying dogs are located and the optimal habitat of Ixodid ticks exist. Cases of *Ehrlicha* were in north, south and east of the West Bank, Palestine (Fig. 1a–c). This is inconsistent with the distribution of livestock and open wilderness with caves and wild vegetation. Kulldorf's saTScan revealed two main statistically significant foci for Ananplasma infection regardless of the host or vector (Fig. 3). These were in Tubas district north of the West Bank and in a village on the hills overlooking the western side of the Jordan Valley. The two statistically significant foci lie on the migration route of birds on the great Syria‐African rift valley extending from East Africa until Syria. The Jordan Valley lying in the middle of the rift is a major point of attraction for these birds to rest in on their way during the spring and autumn annual migration. Although not proved in Palestine, migrating birds have been found to carry infected ticks and transfer tick‐borne diseases from one area to another as found in Sweden (Kuo *et al*. 2017). Another plausible explanation for the two foci is the climate change. The vector‐borne diseases are climate‐sensitive and the vectors have been found to move north as far as Norway, Sweden and Russia as well as to higher altitudes (Jore *et al*. 2011; Andreassen *et al*. 2012; Ostfeld & Brunner 2015). Furthermore, the two areas are considered under‐privileged rural areas with low socioeconomic status and leading extensively active life style of farming and livestock‐raising which again could have contributed to the high disease rate (Campbell‐Lendrum *et al*. 2015). Anyhow, this is far from being a single factor event, but largely multifactorial with complex interactions of several variables such as climate change, environmental change and human behavior.

## Conclusion

The results of this study highlight for the first time the presence of *A. platys* and *E. canis* infection in dogs from Palestine. *A. ovis* is also detected in sheep indicating a potential risk for adverse more serious disease. As part of a comprehensive control strategy, veterinarians in Palestine should put into consideration the presence of *Anaplasma* and *Ehrlichia* during clinical examination of sick animals particularly when clinical signs are compatible with anaplasmosis and ehrlichiosis. The possibility of cross‐border spill out of infection to neighboring countries should trigger regional cooperation to control the diseases. In addition, community and health professional awareness and surveillance system for neglected, yet emerging, zoonotic tick‐borne diseases are recommended to be undertaken at the official level.

## Conflict of interest

The authors declare that the research was conducted without any conflict of interest.

## Ethical statement

The animal owners were verbally informed about the goals of the study and the sampling protocol. All owners gave their verbal informed consent to collect ticks and blood samples from their animals. The study was approved by the ethics committee at the Faculty of Medicine in Al‐Quds University‐Palestine (EC number: ZA/196/013).
